# Protein Interaction and Na/K-ATPase-Mediated Signal Transduction

**DOI:** 10.3390/molecules22060990

**Published:** 2017-06-14

**Authors:** Xiaoyu Cui, Zijian Xie

**Affiliations:** Marshall Institute for Interdisciplinary Research, Marshall University, Huntington, WV 25703, USA; cuix@marshall.edu

**Keywords:** Na/K-ATPase, Src, signal transduction, cardiotonic steroids, drug target

## Abstract

The Na/K-ATPase (NKA), or Na pump, is a member of the P-type ATPase superfamily. In addition to pumping ions across cell membrane, it is engaged in assembly of multiple protein complexes in the plasma membrane. This assembly allows NKA to perform many non-pumping functions including signal transduction that are important for animal physiology and disease progression. This article will focus on the role of protein interaction in NKA-mediated signal transduction, and its potential utility as target for developing new therapeutics.

## 1. Introduction

The Na/K-ATPase (NKA) was discovered by Skou 60 years ago as the molecular machine for pumping Na^+^ and K^+^ across cell membrane [1]. In the early 1970s, several studies revealed the regulatory effects of ouabain on cell growth and gene expression. At that time, these regulatory effects of ouabain were all ascribed to the pump inhibition and the resulted change in intracellular ion concentration [2,3,4]. About 20 years ago, a series of studies conducted first in neonatal cardiac myocytes and subsequently in renal epithelial cells, showed that ouabain could activate a number of cell growth-related pathways, of which many are independent of changes in intracellular ion concentration. These studies have led to a great effort by many laboratories and subsequent demonstration that the NKA actually has many non-pumping functions [5,6]. In this review, we will first look back at our evolved view of NKA in cell biology. We will then give an in-depth discussion of NKA-mediated signal transduction; its role in animal physiology and disease progression; theoretical consideration and experimental evidence of direct protein interactions as the molecular mechanism; and the possibility of targeting such interactions for developing new therapeutics.

## 2. Na/K-ATPase and Active Ion Transport

NKA belongs to the P-type ATPase family. Before Skou discovered NKA in 1957, cell biologists had speculated the existence of such transmembrane machinery for over 100 years. One of the most important early studies was conducted by Carl Schmidt who demonstrated the existence of a Na^+^/K^+^ concentration gradient across cell membrane [7]. This led to the proposal by Rudolf Heidenhain of a “microscopic steamship” laying within the membrane that is capable of maintaining this gradient [8]. Subsequently, several key discoveries paved the way and convinced cell biologists of a principle responsible for transmembrane movement of ions against their concentration gradients. Most notably were the studies by Ernest Overton, showing that muscle cells had active transport mechanism allowing cells to move Na^+^ and K^+^ across cell membrane via the consumption of energy [9,10]. This was confirmed by Heppel and Steinbach in muscle cells using isotopes [11,12,13] and by several groups of U.S. scientists in red blood cells [14,15,16]. Finally, cardiac glycosides were found to be specific inhibitors of such active transport in red blood cells [17], and the requirement of ATP for K^+^ uptake in these cells further supported and linked the transport system to membrane-bound ATPase sensitive to cardiac glycosides [18,19].

**Figure 1:**
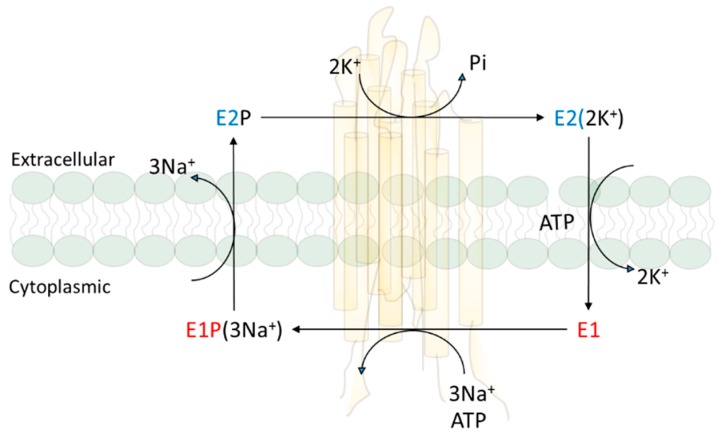
Schematic presentation of Albers–Post reaction mechanism.

At the time Skou discovered NKA, Robert Post had found that the ATPase is responsible for the active transport of three Na^+^ and two K^+^ across the plasma membrane in red blood cells. His subsequent work on the reaction mechanism led to the Albers–Post scheme that is not only true to the NKA, but also applies to other members of P-type ATPase family [20,21,22]. Ion pumping is linked to the cycle of conformational changes. Around the same time, cell biologists and renal physiologists developed a kidney NKA purification protocol, and generated a large number of important mechanistic and cell biological data that refine the structure, reaction mechanism, and cellular regulation of NKA [23,24,25,26]. Importantly, we understand that NKA exists in a dynamic state of conformation equilibrium which was important for its ability to convert ATP hydrolysis to the binding and movement of ions across the plasma membrane as illustrated in Albers–Post reaction mechanism scheme (Figure 1). It also allows the binding of many ligands (chemicals such as cardiotonic steroids that can bind to NKA with high affinity) to the NKA in a conformational state-dependent manner.

NKA, as a large and highly expressed membrane protein complex (most cells contain over one million surface pumps per cell), consists of two noncovalently linked subunits, α and β [27,28]. The α subunit contains ATP and other ligand binding sites, and is considered as the catalytic subunit. The scaffolding function of β subunit is essential for the membrane targeting and full function of the NKA. Four isoforms of NKA have been identified. The existence of different isoforms was first suggested by Michael Marks and Nicholas Seeds in 1987 [29]. They found that ouabain exhibited two distinct inhibition phases of NKA in preparations made from the mouse brain. Subsequently, Sweadner identified at least two isoforms of NKA in membrane preparations from rat brain [30]. The further breakthrough came from the molecular cloning of NKA, first from sheep kidney in 1985 [31], and then the identification of four isoforms from different rat tissues [31,32,33]. Furthermore, studies showed that different isoforms are expressed in a tissue-specific manner [32,33]. The α1 isoform is found in all cells and is prevalent in all epithelial cells. The α2 and α3 isoforms are expressed in skeletal muscle, neuronal tissue, and cardiac myocytes. The α4 isoform is expressed in the testis and regulates sperm motility [27,34]. The sequence identity is about 87% among α1, α2 and α3, while α1 and α4 are 78% identical. Nevertheless, the overall tertiary structure appears to be identical among all isoforms [35].

3D structures of several P-type ATPases including α1 NKA have been resolved [36,37,38,39]. The overall structure of NKA is composed of ten transmembrane helices important for ion binding, occlusion and movement, and three cytosolic domains called the N-domain (nucleotide binding), P-domain (phosphorylation) and A-domain (actuator) that confer ATP hydrolyzing activity. Overall, crystal structures are in agreement with the deduced structures from biochemical studies of the past 60 years. Interestingly, recent resolution of several different CTS-bound NKAs also reveals that although these compounds all inhibit ATPase activity they could actually produce different structure perturbations [40,41].

In short, a lot has been learnt about the structure and function of NKA as an ion pump over the last 60 years. Moreover, we have gradually recognized that the NKA may be engaged in dynamic interaction with other membrane and cytosolic proteins because of the dynamic nature of NKA conformational equilibrium and the large number of NKA in the plasma membrane. Such interactions play at least three different roles in cell biology: (1) dynamic regulation of ionic concentrations including Na^+^, K^+^ and consequently Ca^2+^ by regulating the pumping activity of NKA, the focus of early years of investigation; (2) a key player in cellular signal transduction because of its direct interactions with signaling proteins; and (3) as a signal integrator by organizing specific membrane microdomains and by bridging different affecters and effectors together through its scaffolding function.

## 3. Na/K-ATPase and Signal Transduction

Cardiotonic Steroids (CTS) include plant-derived digitalis such as digoxin and ouabain, and vertebrate-derived aglycones such as bufalin and marinobufagenin (MBG) [42,43]. Digoxin has been used to manage congestive heart failure for over 200 years, and bufalin/MBG are active components in traditional Chinese medicine “Chan Su”. However, the digitalis-specific inhibition of NKA was not recognized until the discovery of NKA in the 1950s [1,17]. In cardiac myocytes, inhibition of NKA activity by CTS increases intracellular Na^+^ concentration, which leads to the accumulation of intracellular Ca^2+^ through functional coupling to the Na^+^/Ca^2+^ exchanger (NCX). Consequently, an increase in Ca^2+^ concentration enhances the contractility of cardiac muscle and causes positive inotropy [42]. In patients with heart/kidney diseases, an increase in endogenous CTS has also been observed [44,45,46].

### 3.1. Na/K-ATPase: More Than a Pump

In addition to its effect on cardiac contraction, CTS were recognized long time ago to play a role in cell growth regulation. In the 1970s, for instance, ouabain at low nM concentrations was found to regulate gene expression and the mitogen-induced differentiation and proliferation in lymphoblasts [3,47,48]. Taking into the consideration that IC_50_ of ouabain is around 50–100 nM for human α1 NKA [49,50,51], such low concentration of ouabain on cell growth is unlikely due to the substantial inhibition of transmembrane movement of ions via the NKA, suggesting additional mechanism being responsible for CTS-induced changes in cell growth. Other than cell growth regulation, picomolar concentrations of MBG can stimulate the synthesis of collagen in human dermal fibroblasts [52].

A series of studies from our laboratory published in the late 1990s and early 2000s revealed that CTS could stimulate protein tyrosine phosphorylation and a number of growth-related pathways in cell type- and tissue type-dependent manner [53,54,55,56,57], which has now been largely confirmed by studies from other laboratories around the world [49,58,59,60,61,62,63,64,65,66,67,68,69,70,71,72,73,74,75]. These new findings suggest NKA as an important signal transducer, and the involvement of protein kinase cascades in the cell growth regulation by CTS rather than the inhibition of ATPase activity. At the time, the importance of NKA-mediated signal transduction had not been fully appreciated and was considered as being “moonlighting”. This is in part because of the following two unresolved albeit important issues. First, since NKA has both pumping and signaling functions, it was difficult to study signaling independent of pumping, especially in cardiac myocytes where NKA is tightly coupled to other membrane transporters such as NCX [42,76,77,78,79,80]. Second, the realization that NKA has no tyrosine kinase or phosphatase activity had raised question as to how binding of ouabain to NKA stimulated protein tyrosine phosphorylation, which was required for the regulatory actions of ouabain on cell growth. Although direct protein interaction was speculated at the time, no experimental evidence, especially the involvement of tyrosine kinase/phosphatase, had been reported in the literature. These issues have driven the next ten years of investigations, and led to our current appreciation of the molecular basis of NKA-mediated signal transduction in cells.

### 3.2. Protein Interaction in Signal Transduction

It is well established that regulated protein interaction is a key to cellular signal transduction. The best studied examples are those of G protein-coupled receptors and receptor tyrosine kinases (RTKs). Extracellular ligand binding to RTKs stabilizes receptor dimerization and causes *trans*-phosphorylation of the receptor. The phosphorylated tyrosine residues provide binding sites for Src homology 2 (SH2) domain- and phosphotyrosine binding domain-containing proteins, and propagate the downstream signaling [81,82].

Interestingly, early studies identified several NKA-interacting proteins such as ankyrin, adducin and FXYD family of proteins [83,84,85]. However, most of these as well as some of the recent studies have focused on the role of such interactions in the regulation of NKA activity and trafficking. For example, FXYD family proteins are expressed in a tissue-specific manner and appear to act as a third subunit of the enzyme [86]. Although it is not required for functional expression of α/β NKA, FXYD proteins interact, and regulate NKA pumping activity [36,87,88,89]. Interestingly, some interactions between FXYDs and NKA are regulated by membrane receptors. For example, FXYD1, also known as phospholemman (PLM), is a principal phosphorylation substrate of c-AMP dependent protein kinase A and of Ca^2+^-phospholipid-dependent protein kinase C at Ser68 (PKA), or at Ser63, Ser68 and Thr69 (PKC) [90,91,92]. Unphosphorylated FXYD1 inhibits NKA through the direct protein interaction [93,94,95]. In addition, a number of signaling proteins has also been identified during the studies of hormonal regulation of NKA trafficking in kidney epithelial cells. For example, dopamine stimulates the recruitment of arrestin, spinophilin, GPCR kinase and 14-3-3ε, to the α1 NKA. In another example, the association of 14-3-3ζ to the α1 subunit facilitates the binding of PI3K to the α1 subunit that subsequently leads to the endocytosis of the NKA [96,97]. Conversely, in response to angiotensin II, adaptor protein-1 attaches to the α1 subunit and facilitates the recruitment of the NKA to the plasma membrane [98]. Functionally, Bcl-2 proteins were also reported directly interacting with NKA [99]. The interactions are critical for control of cell survival and apoptosis. The ratio of pro-survival and pro-apoptotic proteins interacting with NKA may determine NKA function.

**Figure 2:**
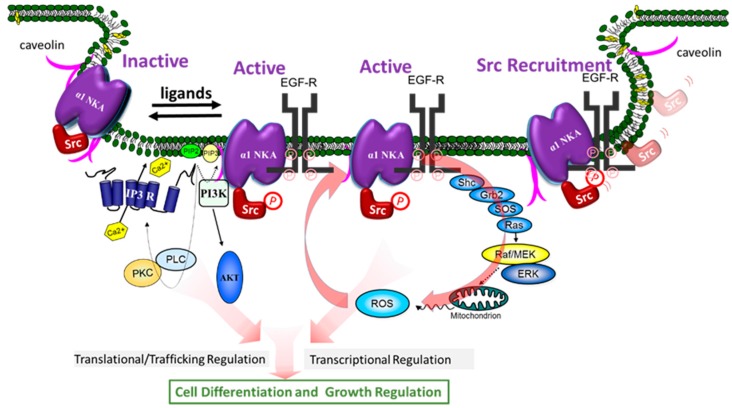
Schematic presentation of NKA-mediated signal transduction through direct protein interactions. EGFR, EGF receptor; PLC, phospholipase C; PKC, protein kinase.

In view of our demonstration that NKA plays a role in signal transduction, the fact that protein interaction is a key to signal transduction has prompted us to ask if NKA is capable of regulating the function of its interacting proteins, and, if so, whether NKA ligands can regulate such interactions, and consequently activate cellular signaling events. The concerted efforts of many over the last ten years have yielded solid evidence that supports this hypothesis. Specifically, these studies have led to the discovery that α1 NKA/Src complex is an important receptor for CTS and other NKA ligands to activate protein/lipid kinase cascades, to generate ROS, and to stimulate Ca^2+^ oscillation in a cell-specific manner (Figure 2). Moreover, new findings have suggested that α1 NKA may regulate such interactions in a conformation-dependent manner. We further speculate that the receptor NKA can actually adapt both active and inactive conformations, and that NKA ligands may stabilize either active (agonists) or inactive (inverse agonists) conformation to exert their regulatory effects on cells (Figure 2). Finally, recent studies have also revealed NKA as a potential signal integrator important for assembling cellular signalosomes and for effective coupling of affecters and effectors.

### 3.3. Src Kinase in NKA-Mediated Signal Transduction

The first clue that Src kinase is important for NKA-mediated signal transduction was from the studies by Haas in early 2000 [56]. Src family kinases are membrane-associated non-receptor tyrosine kinases, and they play an essential role in the signal transduction pathways provoked by many extracellular stimuli such as growth factors, and ligands of G protein-coupled receptors [100]. We and others have shown that α1 NKA regulates Src activity through a conformation-dependent interaction. It also plays an important role in Src targeting through a phosphorylation-dependent mechanism. Binding of CTS to this NKA/Src receptor complex leads to the activation of the associated Src, recruitment of additional Src, and the initiation of the signal transduction processes (Figure 2) [101].

### 3.4. Evidence of NKA/Src Interaction

The following evidence supports the hypothesis that NKA and Src form a functional receptor. First, ouabain and other CTS stimulated protein tyrosine phosphorylation in many different types of cells including cardiac myocytes, smooth muscle and renal epithelial cells to name a few [54,56,59,102,103,104,105,106]. Second, upon ouabain stimulation, Src activation, as evidenced by its translocation from cytosolic fraction to a Triton-insoluble fraction, and an increase in Y418 phosphorylation (pY418) (but not a decrease in Y529 phosphorylation), was one of the earliest events [56]. Moreover, Src inhibitors blocked the ouabain-induced tyrosine phosphorylation and ouabain-activated downstream signal pathways such as ERK. The CTS-induced cell growth effect could also be sufficiently attenuated by Src kinase inhibitors [56,103]. Third, genetic evidence also supports the requirement of Src in ouabain-induced signal transduction because ouabain failed to increase protein tyrosine phosphorylation in SYF cells where Src family kinases are knocked out. On the other hand, rescuing these cells with Src fully restored ouabain-induced signal transduction [107]. Fourth, NKA and Src were co-enriched in caveolar fractions in many cell types [107]. While immunofluorescence imaging analyses confirmed the co-localization of these two proteins, FRET analyses suggested a direct interaction between these two proteins [101]. Further evidence of direct interaction came from co-immunoprecipitation experiments, first reported by Haas et al. [56], then confirmed by many others [59,103,104,105,108,109,110]. However, Kaplan et al. reported the failure of co-immunoprecipitation of NKA with Src in breast cancer cells and questioned whether NKA interacts with Src [111]. Although it remains to be determined, it is important to note that Kaplan lab conducted the immunoprecipitation using a different anti-α1 antibody from other labs. Interestingly, the polyclonal antibody used by Kaplan lab was raised against the fragment of α1 NKA where the putative Src binding site resides, which ironically could provide further support of direct interaction. In addition to immunoprecipitation analyses, GST-fused fragments of intracellular domains of α1 NKA also pulled-down Src from cell lysates, indicating the existence of a direct and specific interaction between these two proteins [101]. Finally, many have demonstrated that the interaction between these two proteins was actually regulated by CTS [56,59,101,103,104,105,108,109,110]. For example, ouabain increased the co-immunoprecipitation of these two proteins, and this increase was sensitive to Src inhibitors [56].

### 3.5. The Identification of Putative Src Binding Sites and the Discovery of NaKtide as a Specific Inhibitor of NKA-Mediated Signal Transduction

The direct interaction between α1 NKA and Src was further demonstrated by co-precipitation studies using purified dog/pig kidney NKA and fully active but unphosphorylated Src [101]. Functionally, this interaction prevented Src Y418 phosphorylation that is required for full activation of Src kinase activity. Using GST pull-down analyses of different cytosolic domains of α1 NKA and functional domains of Src, two putative Src binding sites have been mapped. One is between second cytosolic domain of α1 subunit of NKA and Src SH2 domain. The other one locates in the N domain of α1 that interacts with Src kinase domain. The latter interaction inhibits Y418 phosphorylation. Again, the polyclonal antibody used in the co-immunoprecipitation from Kaplan Lab was directed to this large fragment of α1 NKA. Importantly, ouabain was shown to release the interaction between purified α1 NKA and Src kinase domain, without affecting the binding of SH2 [101,112]. Further mapping of this interaction has identified the 20 amino acid NaKtide sequence in the N domain of α1 NKA being responsible for the direct interaction between α1 and Src kinase domain [112]. The synthesized NaKtide mimics NKA, capable of interacting with and inhibiting Src. Moreover, when NaKtide is made into pNaKtide by adding TAT (13 amino acids) sequence to the N-terminus, it becomes cell permeable. Functional studies demonstrate that pNaKtide also mimics NKA, and is effective in inhibiting NKA-interacting pool of Src in an ATP concentration-independent manner. Consequently, it blocks ouabain-induced activation of Src, ERK and hypertrophic growth in cardiac myocytes [112]. It also specifically suppresses cell proliferation in cancers, whose Src activity is not efficiently inhibited by α1 NKA [113].

### 3.6. Identification of CD2 as an Important Src SH2 Ligand

Although the interaction between Src SH2 domain and NKA α1 is not reduced upon ouabain binding, it is important in the recruitment and targeting of Src. In LLC-PK1 cells transfected with α1 CD2, exogenously expressed CD2 competitively bound to Src kinase in cells and prevented Src from being targeted to various effectors. Therefore, as the exogenous Src SH2 ligand, α1 CD2 increased the global Src activity but blocked Src-mediated pathways including ouabain-induced signal transduction [114]. In short, we and others have generated strong evidence of α1 NKA/Src interaction and demonstrated the importance of such interaction in CTS-induced signal transduction. However, it is important to note that Karlish and his colleagues have questioned such interaction based on their work with purified recombinant human α1 NKA expressed in yeast and Src kinase expressed in bacteria [115]. A major difference is noted between the study from Karlish and our investigation. We used non-phosphorylated Src in our studies whereas Karlish used bacteria-expressed Src that is known to be phosphorylated at both Y418 and Y529 sites. Phosphorylation of these sites affects the activity and binding of Src to other proteins [116]. For example, it is known that SH2 plays an important role in directing the interaction between Src and its partners [117]. This is also true for NKA/Src interaction as we reported recently [114]. The heterogeneous nature of bacteria-expressed Src in both Y418 and Y529 phosphorylation makes it difficult to measure the potential interaction involving the SH2 domain, especially if the interaction has low affinity. To this end, we and others have demonstrated that inhibition of Src by PP2 could abolish ouabain-induced increases in Src binding, reaffirming an important role of Src-mediated phosphorylation and SH2-directed interaction.

### 3.7. The Identification of a Mutant α1 NKA That Pumps but Is Null in Src Interaction

To further verify that NaKtide sequence is engaged in direct interaction with Src, we performed mutagenesis studies [118]. These studies indicated that the N-terminal helical structure of NaKtide is important for the Src binding and inhibition. Moreover, W423, L424, and R427 appear to be in direct contact with Src kinase domain. This conclusion was further supported by mutations (e.g., A420P and A425P) that disrupt the formation of helical structure. Significantly, when A420P or A425P was introduced into full length α1 NKA, we found that the mutated NKA retained full pumping capacity but failed in Src interaction and regulation [118]. Now, we have two mutant α1 NKA that work as a pump but not a signaling receptor.

### 3.8. NKA/Src Interactions Are Isoform-Specific

**Figure 3:**
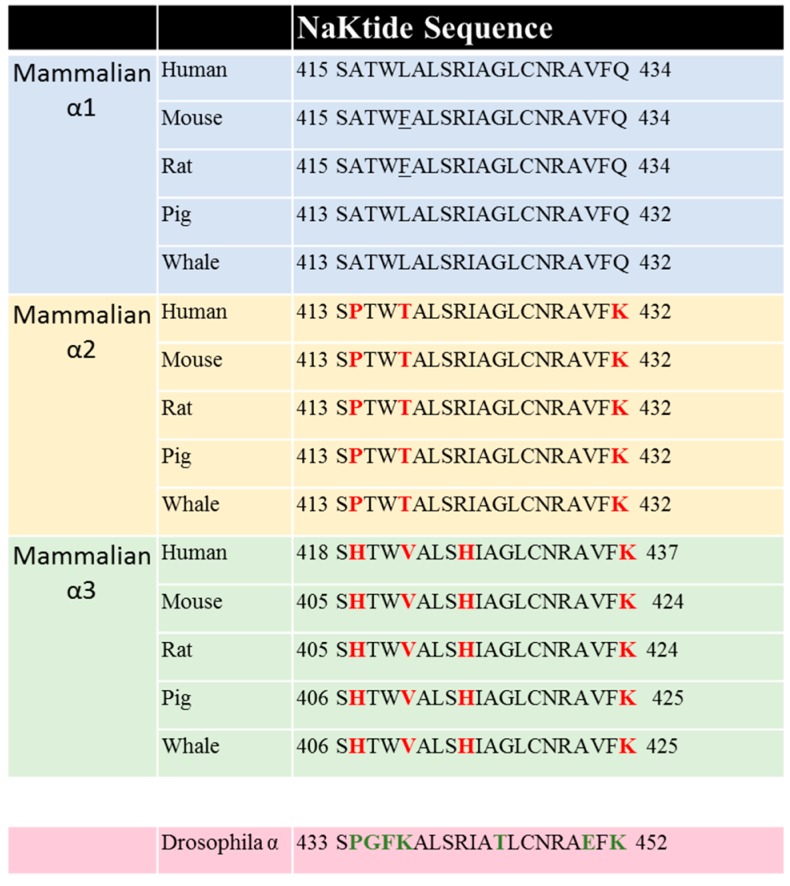
Comparison of NaKtide sequences among different species in α1, α2 and α3 subunits. The amino acids being different from mammalian α1 are marked in red. Data are from NCBI data base.

Sequence comparison shows that the NaKtide sequence is highly conserved in mammalian α1 NKA. However, the corresponding NaKtide sequences in α2 and α3 isoforms are different from that in α1. Interestingly, these differences are also conserved in both α2 and α3 sequences (Figure 3). In view of the importance of NaKtide sequence in α1 NKA-mediated Src interaction, we generated α2 and α3-expressing mammalian cells using a knock-down and rescue protocol, and demonstrated that both α2 and α3 NKA isoforms lack Src-interacting capacity [119,120]. As such, they do not carry Src-dependent signal transduction upon ouabain binding. However, α3 isoform differs from α2 because it does signal in a Src-independent manner [119]. These new findings reveal a major functional difference among three NKA isoforms, and re-enforces the importance of Src binding capacity of α1 NKA in the regulation of cell signal transduction. In addition, the observed difference in their ability to conduct signal transduction among three isoforms provide strong evidence that CTS-induced activation of protein kinases is unlikely due to changes in intracellular ATP secondary to the inhibition of the pumping as advocated by some [121,122]. Finally, Lingrel lab and others have generated strong evidence that different NKA isoforms do exert distinct regulation of animal physiology (e.g., muscle contraction). Therefore, our new findings also suggest the importance of the lack of Src binding for α2 and α3 specific cellular singling functions.

### 3.9. NKA/Src Complex as a Receptor

Mechanistic investigations of last fifteen years have revealed a novel molecular mechanism of NKA-mediated signal transduction. As illustrated in Figure 2, the receptor NKA interacts with several proteins to perform cell-specific signal transductions including Raf/MEK/ERK, PLC/PKC, PI3K/Akt, and Ca^2+^ signaling and the generation of ROS. One of the most important signaling partners *trans*-activated by NKA/Src receptor complex is EGF receptor, which is recruited and phosphorylated at several phosphorylation sites other than its major phosphorylation site Y1173 when cells are exposed to CTS [56]. The activated EGF receptor then recruits the adaptor protein Shc, which in turn binds the protein complex Grb2 and SOS. SOS is a guanine nucleotide exchange factor that activates Ras by exchanging GDP for GTP. Activated Ras then stimulates Raf/MEK and p42/44 ERK cascade [56,57]. Activation of this cascade by CTS appears to occur in most of cell types [57,59,104,109,123]. It is of interest to note that activated EGFR is also a critical element in the signal transduction networks of cytokines, H_2_O_2_, and pathways utilizing G protein-coupled receptors (51). However, the number of membrane α1 NKA in most of cells is at least over 100 times of G protein-coupled receptors. Thus, it is reasonable to speculate that α1 NKA may regulate the Src-dependent signaling pathways of G protein-coupled receptors. Moreover, in view of a critical role of EGFR in cancer, it would be of great importance to further dissect α1 NKA-mediated regulation of EGFR and its potential role in cancer biology.

Several important features of this newly appreciated signaling mechanism are worthy of further discussion. First, the activation of protein and lipid kinase cascades and the generation of second messengers ensure the formation of a positive feed forward loop that could amplify CTS-provoked signal transduction, and also allow signal diversification, transcriptional and translational regulation of gene expression [52,53,57]. This is best exemplified by the recruitment of additional signaling partners into the receptor complex [57,107,124], and by the ROS-induced signal amplification (Figure 2) [55,125,126]. In accordance, it explains how endogenous CTS could exert profound physiological effects at concentrations well below 1/100th of IC_50_ [127]. For example, it has been reported that ouabain at 10 to 100 nM was sufficient to stimulate mouse or rat cardiac fibroblasts, resulting in increased collagen production [52,128]. Similarly, such low concentrations of CTS were found to elicit Ca^2+^ oscillation in both mouse and rat kidney epithelial cells where only the ouabain-resistant α1 NKA is expressed [129]. It is also important to point out that the fetal bovine serum we all use in our cell culture may contain sufficient amount of CTS to promote cell growth [130]. Finally, signal amplification similar to these in rodents has also been observed in human cells [52].

Second, because NKA contains a large number of motifs both intracellularly and extracellularly, it would not be a surprise that NKA could perform much more regulatory function than those outlined in the scheme (Figure 2). Moreover, it is likely that many of these pathways could cross-talk to each other and exert cell-specific regulation depending on the context of available signaling constitutes. This is exemplified by the fact that NKA could regulate PI3K signaling in Src-knock out cells, whereas inhibition of Src also attenuates ouabain-induced PI3K signaling in normal cells [131,132,133,134]. Similarly, Src is also involved in ouabain-induced Ca^2+^ oscillation by affecting the interaction between IP3 receptor and α1 NKA in renal epithelial cells [129].

Third, this scheme provides a framework to begin addressing the role of NKA-mediated signal transduction in animal physiology. To this end, recent animal studies have demonstrated the importance of this signaling mechanism in wild array of physiological processes including renal salt handling, vascular activity, cardiac growth, and embryonic development to name a few [106,135,136,137,138,139,140,141,142,143]. These new findings call for the need of re-examination of CTS physiology and exploring the potential new pharmacology of exogenous CTS [144]. In the past, most pharmacological studies of CTS were focused on their ability to inhibit NKA. As such, they were used as NKA inhibitors to increase myocardial contractility. Even in this application, clinical studies have demonstrated that the use of lower, but not higher, doses of digoxin is associated with a decrease in mortality in patients with congestive heart failure [145]. Interestingly, recent studies have shown that the activation of NKA signaling, but not inhibition of cellular pump capacity, by CTS is capable of protecting the heart from ischemia/reperfusion injury in rats [146,147,148]. Furthermore, CTS at doses lower than 1/100th of IC_50_ of NKA activity are effective stimuli of collagen synthesis, suggesting the potential use of these compounds in skin care and wound healing [52,128]. It is equally important to recognize that CTS could also inhibit cell growth in a wide variety of cancer cell lines such as prostate, lung, colon cancer cells and neuroblastoma cells by stimulating several different pathways, including apoptosis and autophage-related processes [61,62,66,70,71,104,149,150,151]. On the other hand, endogenous CTS may play an important role in the pathogenesis of autosomal dominant polycystic kidney disease (ADPKD) by activating Src/EGF receptor/ERK pathways [109,152,153].

Finally, it has been reported that the endocytosis of NKA/Src receptor complex, like many other membrane receptors, is stimulated by its ligands such as CTS [131,154,155]. This occurs via clathrin-coated pits, early and late endosomes, and depends on the activation of Src and PI3K. Although it remains to be further investigated, it is conceivable that CTS-induced endocytosis of receptor NKA/Src could represent a pathway of signal termination. Of course, it might also generate an effective way of communication with intracellular compartments during the signal transduction process [156].

## 4. Conformation-Dependent Regulation of Src by α1 NKA, a New Hypothesis

The essence of receptor-mediated signal transduction is the intrinsic ability of a receptor to adapt both active and inactive conformational states [157,158]. Several important but seeming un-related studies have led us to test this important concept (hypothesis) in NKA-mediated signal transduction. The first clue was actually from the studies of purified NKA/Src interaction. As reported by Tian et al. [101], purified kidney α1 NKA inhibited Src Y418 phosphorylation, and that addition of ouabain restored Y418 phosphorylation only in the presence of α1 NKA. Interestingly, while vanadate also inhibited ATPase activity of α1 NKA, it showed minimal effect on Y418 phosphorylation at concentrations that produced similar degree of NKA inhibition as ouabain. Most significantly, ouabain was able to further stimulate Y418 phosphorylation in the presence of vanadate that caused complete inhibition of α1 NKA. Because it is known that vanadate facilitates ouabain binding to the α1 NKA, these findings suggest that α1 NKA may interact and regulate Src activity in a conformation-dependent manner. The second line of evidence came from studies of xanthone derivatives. These compounds are potent and specific inhibitors of α1 NKA. However, they show no ouabain-like effect on α1 NKA/Src interaction [159]. This led to studies of Ye et al., testing whether α1 NKA/Src interaction can be modeled on the Albers–Post scheme [134]. By using well-characterized conformation stabilizing chemicals as well as α1 NKA mutant defective in conformation transitions, we find strong evidence that α1 NKA, like G protein-coupled receptors, can adapt both active and inactive conformations to interact and regulate Src. This new framework, taken together with our appreciation of Albers–Post scheme, has led us to deduce that α1 NKA may represent a broad cell signaling mechanism. As such, many ligands of α1 NKA, including CTS and intracellular/extracellular ions, may alter cellular signal transduction through a Src-dependent process (Figure 2).

### 4.1. NKA/Src/ROS Loop and Disease Progression

ROS participates in various cellular activities [160,161,162,163,164,165]. Our early studies demonstrate that ouabain stimulates ROS generation in a Ras-dependent way via NKA/ Src signaling [55,166]. On the other hand, modification of α1 NKA by ROS has been well documented [125,167,168,169,170,171,172], and such modification by ROS can directly alter the conformation states of α1 NKA [173]. Our new appreciation of α1 NKA/Src signaling mechanism has led to several studies of whether ROS can work similar to CTS on NKA/Src complex. These studies have led to the following observations. First, an increase in H_2_O_2_ generation is sufficient to cause the activation of Src and ERK, and stimulated α1 NKA endocytosis in LLC-PK1 cells. Disruption of NKA/Src interaction by either pNaKtide or by the expression of Src-interaction null mutants (A420P) abolishes H_2_O_2_-induced Src/ERK activation [126]. On the other hand, ouabain stimulates the generation of ROS, which results in direct carbonylation of Pro 222 and Thr 224 in the α1 subunit of NKA [125]. Moreover, inhibition of the carbonylation by anti-oxidants attenuates ouabain-induced activation of protein kinase cascades. Thus, it is proposed that NKA/Src and ROS form a signal amplification loop allowing not only CTS but also ROS to generate signals from the NKA.

In view of the well-established role of ROS stress in the progression of many chronic diseases, we and others have recently explored whether the newly appreciated NKA/Src/ROS loop is essential for un-regulated ROS signaling. These studies have demonstrated that this signaling loop is indeed activated and plays an important role in the development of atherosclerosis, renal inflammation-induced tissue damage, and metabolic syndrome as well as uremic cardiomyopathy [136,139,174].

### 4.2. NKA/Src Interaction as a Drug Target

The rationale for targeting NKA-mediated signal transduction to develop new therapeutics has been discussed [6,43,144]. Recent in vitro and in vivo studies have demonstrated the feasibility of targeting NKA/Src interaction, and effectiveness of an inhibitor, pNaKtide, of this interaction as a potential therapeutics of cardio-renal diseases and metabolic syndrome. As discussed above, pNaKtide is composed of NaKtide sequence (20 amino acid peptide) from human α1 NKA and a TAT leader (13 amino acids peptide). The TAT leader is the so-called cell penetrating peptide that helps large molecules to across cell membrane. pNaKtide not only readily passes cell membrane, it resides, similar to α1 NKA, in the plasma membrane, making it highly specific as an inhibitor of NKA/Src complex. Moreover, it is potent, as 0.1 to 1 μM is sufficient to completely block CTS- or ROS-induced signal transduction in cell cultures [112,126]. It also has a good safety profile as no cellular toxicity was observed up to 20 μM in three different cell lines. Remarkably, it is readily taken up in vivo by the heart, kidney, liver and fat tissues, and shows a plasma membrane distribution as well [140,175,176]. Significantly, pNaKtide was effective in blocking ROS amplification and α1 NKA-mediated signal transduction in animals fed with high fat diet, and consequently attenuated metabolic syndrome [140]. Moreover, recent studies have further demonstrated its effectiveness as an inhibitor of ROS amplification and NKA/Src signaling in animal model of chronic kidney failure, Western diet-induced liver damage and atherosclerosis [175,176]. For example, in animal models of uremic cardiomyopathy induced by 5/6 nephrectomy, it not only prevented cardiac hypertrophy and fibrosis, but also improved cardiac function and hematocrit. Remarkably, it was also capable of reversing cardiac lesions in a dose-dependent manner [140,175].

## 5. Conclusions and Perspectives

Studies from many laboratories of the past 20 years have documented that NKA has an ion-pumping independent receptor function that confers a ligand-like effect of CTS on protein/lipid kinases, intracellular Ca^2+^ oscillation and ROS generation. Direct protein interactions between NKA and its partners are responsible for this newly appreciated signaling mechanism. Meanwhile, our appreciation of this signaling mechanism has also evolved from “moonlighting” to an essential pathway in animal physiology and disease progression. It is important to recognize that the aforementioned investigations only mark the beginning of a fascinating field. In addition to the continued effort of many in defining the molecular mechanism of NKA-mediated signal transduction, isoform-specificity and identifying cell/tissue-specific signalosomes, the following two areas of research may further advance our understanding of NKA. First, efforts have been and will continue to be made to generate new animal models including transgenic animals with specific defect in NKA signaling and tool drugs targeting NKA/Src or NKA/other partner interactions. These new animal models and tool drugs will help advancing our understanding of NKA-mediated signal transduction in animal physiology and disease progression, provide further validation of NKA-mediated protein interaction as a druggable target, and generate lead candidates for the development of clinically useful drugs.

Second, NKA, as discussed, interacts with many proteins. Unlike other membrane receptors, it is highly expressed in most of cells. As such, it may also work as an important scaffold. As an example, it is of interesting to look at the interaction of α1 NKA with caveolin-1, and a Src-dependent interplay among the α1 NKA, caveolin-1 and cholesterol. Both caveolin-1 and cholesterol are important structural components of caveolae that are flask-shaped vesicular invaginations on the plasma membrane [154,177,178,179]. Caveolae are known to play an important role in cellular signal transduction. The α1 subunit of NKA contains a highly conserved caveolin binding motif at the N-terminus, and brings caveolin-1 to its regulatory kinase Src [107,179]. Reduction in the expression of α1 NKA stimulates Src, resulting in an increase in caveolin-1 Y14 phosphorylation. This leads to the reduction of membrane caveolin-1 and cholesterol, and consequently a decrease in the number of caveolae [179,180]. On the other hand, reduction of membrane cholesterol can activate Src in a α1 NKA-dependent manner, leading to an increase in the endocytosis of α1 NKA. Thus, this Src-dependent interplay may establish a highly efficient feed-forward mechanism that detects the change in cellular cholesterol and/or α1 NKA, and then alters the structure and function of the plasma membrane [107,123,178,179,181,182,183,184,185]. In accordance, studies have shown that α1 NKA/caveolin-1 interaction is not only essential for CTS-induced activation of protein/lipid kinase cascades, but also for α1 NKA to interact with other signaling proteins. One of these is IP3 receptor (Figure 2). This latter interaction may also depend on other scaffolding proteins such as ankyrin. Nevertheless, the interaction between caveolar α1 NKA and ER IP3R allows the formation of an efficient Ca^2+^ signaling machine by tethering affecters (e.g., membrane receptors), signal transducer (e.g., Src and phospholipase C) and effectors (e.g., Ca^2+^ channels) together. Consequently, α1 NKA is necessary not only for CTS but also for purinergic stimulation of Ca^2+^ oscillation [107,124,186]. Clearly, much remains to be learned about the potential role of α1 NKA as scaffold and its interplay with other receptors in animal physiology and disease progression, which could open up new opportunities for the discovery of other NKA-specific drug targets.
